# TIM-1 Augments Cellular Entry of Ebola Virus Species and Mutants, Which Is Blocked by Recombinant TIM-1 Protein

**DOI:** 10.1128/spectrum.02212-21

**Published:** 2022-04-06

**Authors:** Min Zhang, Xinwei Wang, Linhan Hu, Yuting Zhang, Hang Zheng, Haiyan Wu, Jing Wang, Longlong Luo, He Xiao, Chunxia Qiao, Xinying Li, Weijin Huang, Youchun Wang, Jiannan Feng, Guojiang Chen

**Affiliations:** a State Key Laboratory of Toxicology and Medical Countermeasures, Institute of Pharmacology and Toxicology, Beijing, China; b Inner Mongolia Key Lab of Molecular Biology, School of Basic Medical Sciences, Inner Mongolia Medical University, Hohhot, China; c Division of HIV/AIDS and Sex-transmitted Virus Vaccines, National Institutes for Food and Drug Control, Beijing, China; University of Sussex

**Keywords:** Ebola virus, recombinant TIM-1 protein, variant surface glycoprotein

## Abstract

Ebola virus, a member of the *Filoviridae* family, utilizes the attachment factors on host cells to support its entry and cause severe tissue damage. TIM-1 has been identified as a predominant attachment factor via interaction with phosphatidylserine (PS) localized on the viral envelope and glycoprotein (GP). In this study, we give the first demonstration that TIM-1 enhances the cellular entry of three species of Ebola virus, as well as those harboring GP mutations (A82V, T544I, and A82V T544I). Furthermore, two TIM-1 variants (i.e., TIM-1-359aa and TIM-1-364aa) had comparable effects on promoting Zaire Ebola virus (EBOV) attachment, internalization, and infection. Importantly, recombinant TIM-1 ectodomain (ECD) protein could decrease the infectivity of Ebola virus and display synergistic inhibitory effects with ADI-15946, a monoclonal antibody with broad neutralizing activity to Ebola virus. Of note, EBOV strains harboring GP mutations (K510E and D552N), which were refractory to antibody treatment, were still sensitive to TIM-1 protein-mediated impairment of infectivity, indicating that TIM-1 protein may represent an alternative therapeutic regimen when antibody evasion occurs.

**IMPORTANCE** The viral genome has acquired numerous mutations with the potential to increase transmission during the 2013-to-2016 outbreak of Ebola virus. EBOV strains harboring GP mutations (A82V, T544I, and A82V T544I), which have been identified to increase viral infectivity in humans, have attracted our attention. Herein, we give the first report that polymorphic TIM-1 enhances the infectivity of three species of Ebola virus, as well as those harboring GP mutations (A82V, T544I, and A82V T544I). We show that recombinant TIM-1 ECD protein could decrease the infectivity of Ebola virus with or without a point mutation and displays synergistic inhibitory effects with ADI-15946. Furthermore, TIM-1 protein potently blocked cell entry of antibody-evading Ebola virus species. These findings highlight the role of TIM-1 in Ebola virus infection and indicate that TIM-1 protein represents a potential therapeutic avenue for Ebola virus and its mutated species.

## INTRODUCTION

Ebola virus is a nonsegmented, single-stranded, negative-sense linear RNA virus belonging to the *Filoviridae* family (1). Of five identified subspecies of Ebola virus, Zaire Ebola virus (EBOV), Bundibugyo Ebola virus (BDBV), and Sudan Ebola virus (SUDV) were the primary subspecies causing severe hemorrhagic fever with a high mortality rate in humans (2–4). Ebola virus glycoprotein (GP) is a surface viral protein and is responsible for viral receptor binding and cell entry. Recent studies have shown that EBOV strains harboring two GP mutations—alanine to valine at position 82 (A82V) and threonine to isoleucine at position 544 (T544I)—may cause an increase in viral infectivity in humans (5–11).

Ebola virus entry into host cells requires interaction with various cell surface molecules (12). Upon internalization into low-pH endosomes, the GP1 is proteolyzed by cathepsins B and L, which facilitates its recognition by endosome-expressing Niemann-Pick C1 (NPC1) and leads to GP2-dependent fusion of the viral and host membranes (13, 14). Several attachment factors enhance Ebola virus cell entry, among which T-cell Ig mucin domain 1 (TIM-1) is a predominant factor through engagement with phosphatidylserine (PS) exposed on the surface of EBOV envelope as well as the receptor-binding domain (RBD) (15–20). TIM is a type I transmembrane glycoprotein with three functional family members (TIM-1, TIM-3, and TIM-4). Its extracellular domain is relatively conserved, consisting of an amino-terminal immunoglobulin-like domain (IgV) and a mucin-like domain (MLD) (21, 22). The IgV domain binds to PS on the outer leaflet of the viral membrane through an IgV domain binding pocket, playing a critical role in Ebola virus entry into the cell (16, 20, 23, 24). However, it remains to be determined if this entry-promoting function of TIM-1 is also available to other Ebola virus species and those point-mutated species. In addition, TIM-1 polymorphisms have been shown to be involved in different susceptibilities to infection by a series of virus (25–29). The role of two major forms of TIM-1 (TIM-1-359aa and TIM-1-364aa) in regulating Ebola virus attachment, internalization, and fusion, however, is still elusive.

Although no licensed therapeutics against Ebola virus are available to date, antibodies represent promising postexposure therapies that exhibit highly neutralizing activity *in vitro* and *in vivo*, such as ADI-15946 and MIL77-2 (30–34). ADI-15946 recognizes the GPs on the surface and has pan-neutralizing capacities to EBOV, BDBV, and SUDV (30). MIL77-2 is a humanized monoclonal antibody (MAb) that is based on chimerized MAb 4G7, which is one member of the antibody cocktail ZMAb (34, 35). Unfortunately, in spite of the pronounced protection achieved, mutations in the viral glycoprotein occur posttreatment with these antibodies. Single-amino-acid changes of GP (K510E and D522N) confer complete resistance to ADI-15946 and MIL77-2, respectively (30, 36). New strategies need to be developed to overcome antibody evasion, which is frequent during viral transmission and treatment (8, 11, 37–39).

In this study, we sought to determine the role of TIM-1 and its variants in promoting cell entry of several types of Ebola virus. Our results show that TIM-1 significantly augments infection of Ebola virus and those mutated species. Recombinant TIM-1 protein could impede the cellular entry of Ebola virus, especially those antibody-evading species.

## RESULTS

### Ectopic expression of TIM-1 enables enhanced Ebola virus cell entry.

**FIG 1:**
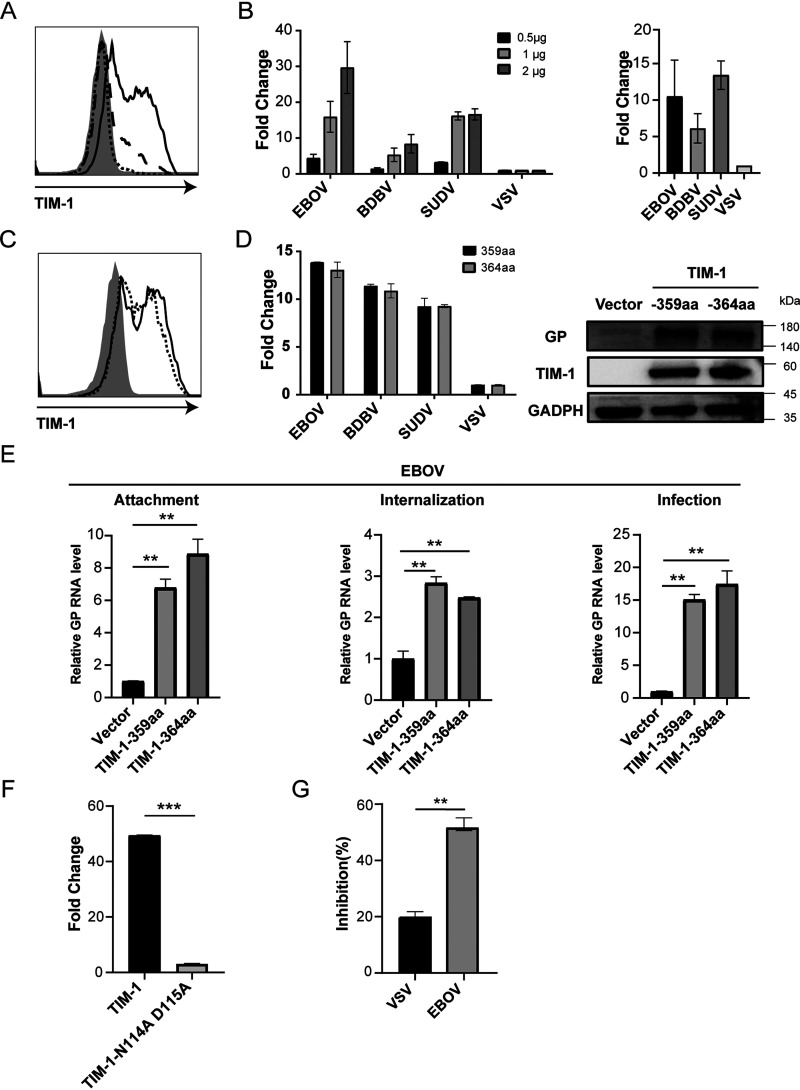
Ectopic expression of TIM-1 enhances Ebola virus cell entry. (A) Surface TIM-1 expression at 36 h following transfection. 293T cells were transfected with 2 μg of empty plasmid (solid gray histogram) or increasing amounts of TIM-1 (black dotted line, 0.5 μg; black dashed line, 1 μg; black solid line, 2 μg). (B) Cell entry of the transfected cells by pSG3.Δenv.cmvFluc (left panel) and VSV-ΔG* (right panel) bearing EBOV, BDBV, SUDV, and VSV GPs. Relative luciferase intensity was detected at 48 h following infection. Data are the fold increase of viral entry into TIM-1-expressing cells compared with cells transfected with empty vector. VSV-pseudotyped virus was used to infect 293T cells transfected with 2 μg TIM-1 and empty vector. (C) Polymorphic TIM-1 expression at 36 h following transfection with 2 μg TIM-1-359aa and TIM-1-364aa. (D) Cell entry of transfected cells by pSG3.Δenv.cmvFluc bearing EBOV, BDBV, SUDV, and VSV GPs (left panel) and cell entry of transfected cells by pSG3.Δenv.cmvFluc bearing EBOV GP by the Western blot method, with GP detected by monoclonal antibody mab114 (right panel). (E) TIM-1 enhances EBOV attachment, internalization, and infection. 293T cells were transfected with an empty vector or plasmids encoding polymorphic TIM-1 for 36 h. EBOV pseudovirus was incubated with cells at 4°C for 30 min and washed with PBS three times (attachment, left panel). After being washed with PBS to remove unbound EBOV particles, cells were transferred to 37°C for 2 h to allow virus internalization and treated with proteinase K to remove uninternalized virions (internalization, middle panel), and then cells were directly challenged with EBOV for 48 h (infection, right panel). Total RNA was extracted and used for quantification of EBOV GP by qRT-PCR. (F) Function comparison of TIM-1 and TIM-1 bearing mutations in the PS binding pocket (TIM-1-N114A D115A) in EBOV cell entry. (G) Function of annexin V to compete for cellular entry of pSG3.Δenv.cmvFluc bearing EBOV and VSV GPs. **, *P < *0.01; ***, *P* < 0.001.

First, we determined the role of TIM-1 in cell entry of Ebola virus. 293T cells, which endogenously do not express TIM-1, were transiently transfected with human TIM-1 expression plasmid at indicated doses (Fig. 1A). Thereafter, these TIM-1-expressing cells were infected with three species of pseudotyped Ebola virus (EBOV, BDBV, and SUDV), whose glycoprotein (GP) was constructed into an HIV vector. As previously described (17, 20), ectopic expression of TIM-1 on the cell surface indeed led to significantly enhanced infectivity of Ebola virus in a dose-dependent manner as studied here (Fig. 1B). To further address this issue, we evaluated TIM-1’s role in promoting Ebola virus entry by using the VSV-ΔG* vector. The results showed that vector replacement did not affect the ability of TIM-1 to augment viral infectivity (Fig. 1B). Given that recent studies have indicated that the polymorphism of TIM-1 was associated with viral susceptibility to host cells (25–29), we asked whether TIM-1 polymorphism was also involved in cell susceptibility to Ebola virus. Two TIM-1 variants (TIM-1-359aa and TIM-1-364aa) were introduced into 293T cells, respectively, with equal levels of expression on the cell surface (Fig. 1C and D). As a result, both TIM-1-359aa and TIM-1-364aa enabled the enhancement of infectivity among three pseudotyped viruses to comparable extents (Fig. 1D). Similar effects were observed when cellular entry of EBOV was detected at the stages of adherence and internalization, respectively (Fig. 1E).

As mutations in the PS binding pocket of TIM-1 from asparagine to alanine at position 114 and aspartic acid to alanine at position 115 (N114A and D115A, respectively) have been reported to abrogate the engagement of TIM-1 with PS (18, 22), we further examined the function of a TIM-1 double mutant (N114A D115A). We found that the introduction of this double mutation remarkably impaired the entry-enhancing ability of TIM-1 (Fig. 1F). To determine whether TIM-1 enhancement of Ebola virus entry was PS dependent, EBOV was preincubated with annexin V, a PS-binding substrate, prior to infection of TIM-1-expressing 293T cells. The results showed that annexin V treatment significantly reduced EBOV infection (Fig. 1G). Overall, these data suggest that TIM-1 augments the infectivity of Ebola virus, at least in part, via the engagement with PS.

### TIM-1 could further enhance mutated species (A82V and A82V T544I) entry.

**FIG 2:**
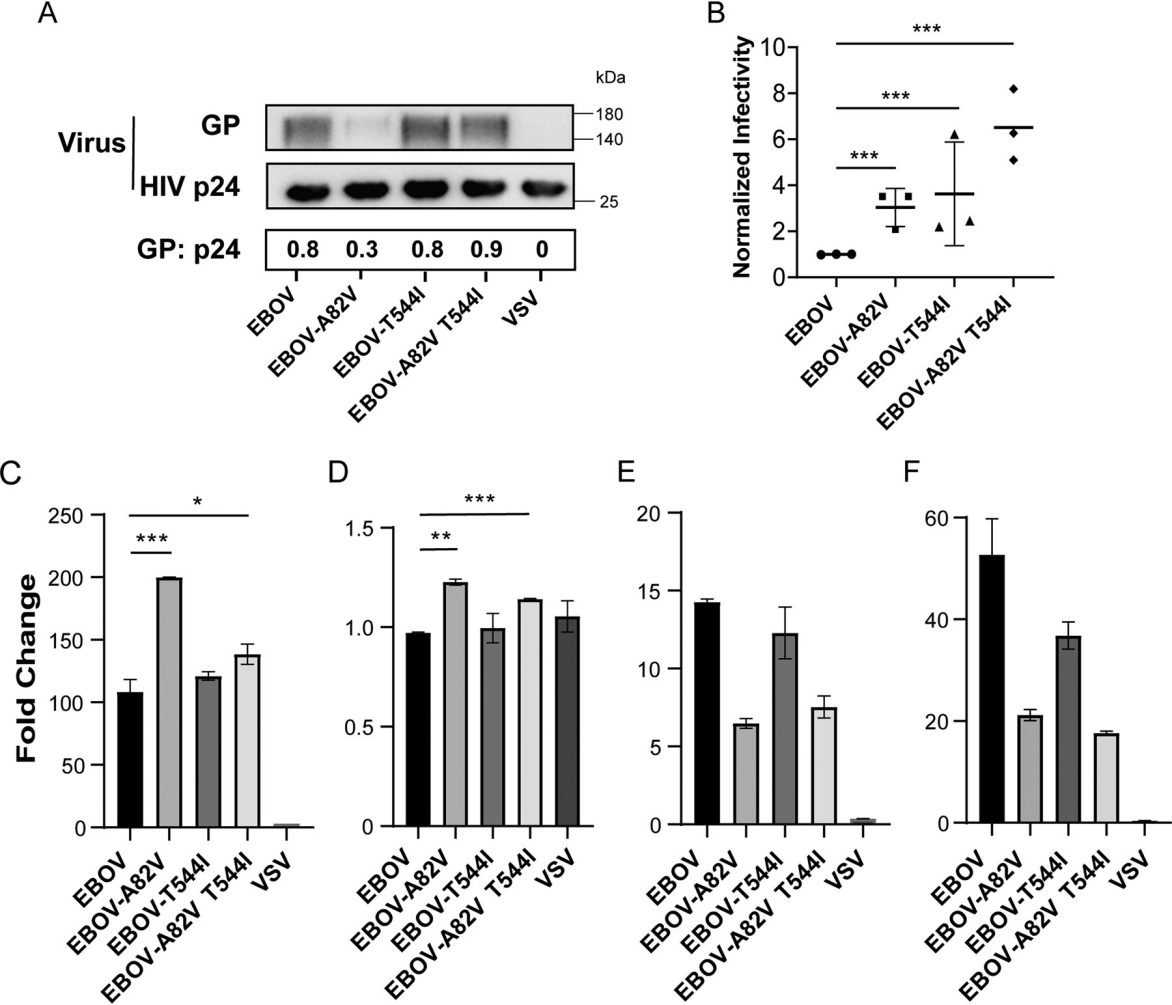
EBOV strains bearing variants of GPs that elicit enhanced infectivity are enhanced by TIM-1 expression. (A) Western blots of lentiviral particles pseudotyped with variant EBOV GPs probed with mab114. The ratio of GP to p24 (GP: p24) was calculated by the Chemiscope 6000 analysis system. In panels B, C, D, and E, the GPs present on virions used here were normalized according to the results of Western blots. (B) Cell entry of the 293T cells by EBOV and EBOV bearing variant GPs. Data points represent independent experiments with three independent viral stocks; each data point represents a normalized transduction. Data are the fold increase of viral entry into TIM-1-expressing (C), Axl-expressing (D), DC-SIGN-expressing (E), and LSECtin-expressing 293T cells (F) compared with vector-expressing 293T cells. *, *P < *0.05; **, *P* < 0.01; ***, *P* < 0.001.

We wondered about the effect of TIM-1 on cell susceptibility of EBOV harboring GP mutations (A82V, T544I, and A82V T544I). The p24 levels were detected by enzyme-linked immunosorbent assay (ELISA) quantitation kit (data not shown), and the amounts of ancestral versus variant GPs present in our EBOV GP-pseudotyped virus were compared by Western blots using a monoclonal antibody, mab114 (Fig. 2A). Viral particles were adjusted to the equivalent level according to viral GP amounts and added to the cells. Compared with parental EBOV, EBOV harboring GP mutations (A82V, T544I, and A82V T544I) enhanced viral entry into 293T cells (Fig. 2B). Furthermore, TIM-1 overexpression rendered a significant increase in the infectivity of EBOV harboring GP mutations (A82V and A82V T544I) (Fig. 2C). Except for TIM-1, Axl is also well demonstrated as a PS receptor to mediate viral entry (40–44). Non-PS attachment factors associated with EBOV viral entry include C-type lectins such as DC-SIGN, DC-SIGNR, L-SIGN, and LSECtin (45–48). In parallel, another PS receptor, Axl, also had entry-enhancing function in these mutated species, although its function was weaker than that of TIM-1 (Fig. 2D). In contrast, non-PS receptors DC-SIGN and LSECtin had no effects (Fig. 2E and F). These data indicate that the enhancement of infectivity of these mutated species is not TIM-1-specific, but PS receptor dependent.

### Recombinant TIM-1 protein competes for Ebola virus cell entry by its IgV domain engagement.

**FIG 3:**
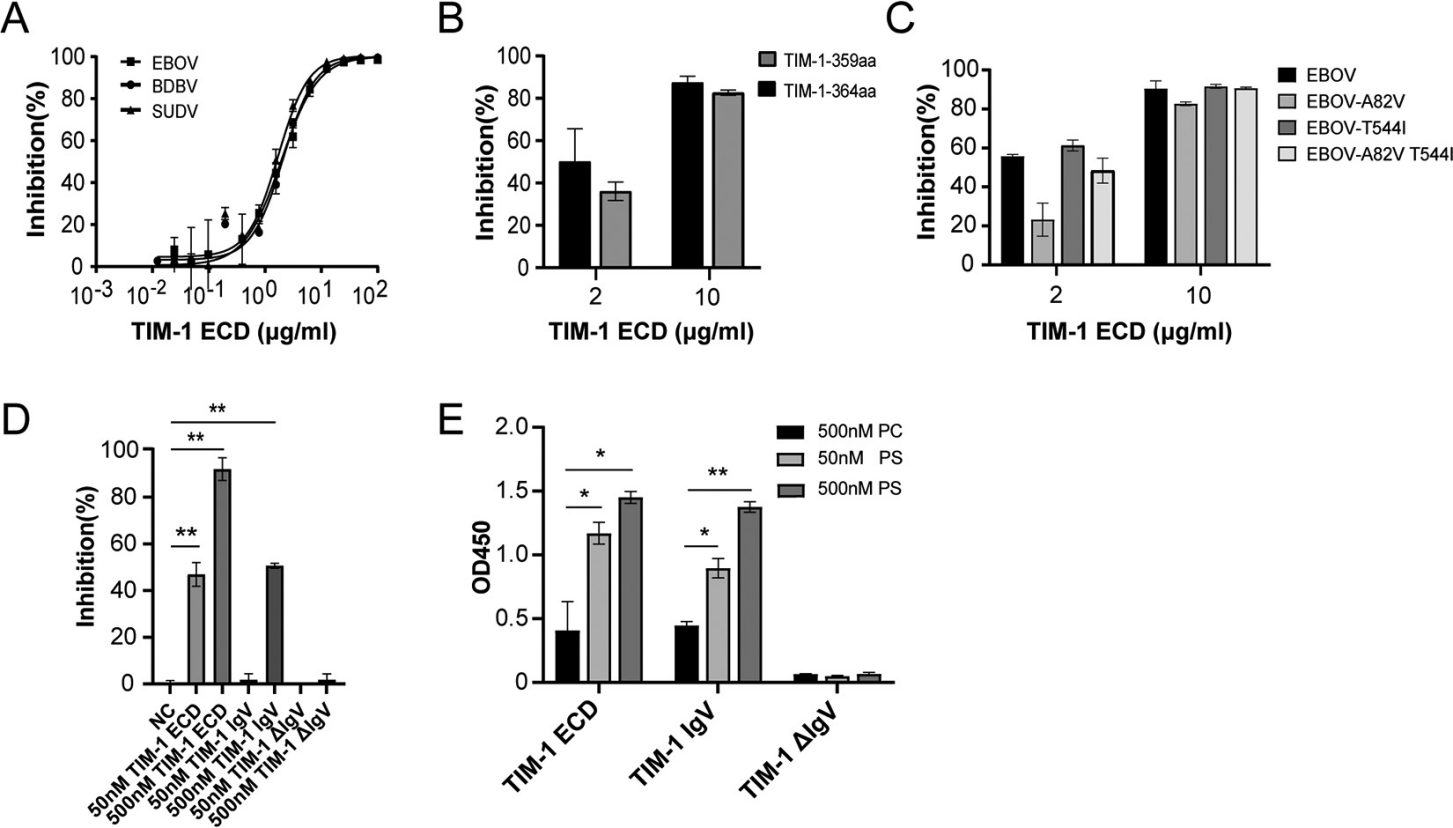
Identification functional properties of TIM-1 protein. TIM-1 proteins inhibit Ebola virus cell entry. (A) Neutralization of EBOV, BDBV, and SUDV by dilutions of TIM-1 ECD. (B) TIM-1 ECD competes for EBOV entry to 293T cells that were transfected with polymorphic TIM-1. (C) TIM-1 ECD competes for EBOV and mutated EBOV cell entry. (D) Function of TIM-1 ECD, TIM-1 IgV, and TIM-1 ΔIgV protein to compete for EBOV entry. (E) Binding affinity of recombinant TIM-1 ECD, TIM-1 IgV, and TIM-1 ΔIgV to PS and PC. *, *P < *0.05; **, *P* < 0.01.

Given the entry-promoting function of TIM-1 described above, we sought to determine whether the recombinant ectodomain of TIM-1 could be a cargo to impede Ebola virus entry. To this end, recombinant TIM-1 (364 amino acids [aa]) ECD protein with hexahistadine tag at the C terminus was prepared. The results showed that TIM-1 protein actively inhibited cell entry of Ebola virus, and the inhibitory effects of TIM-1 protein on EBOV entry were similar to those on the other two Ebola virus species (Fig. 3A). Furthermore, TIM-1 protein administration abrogated TIM-1-364aa and -359aa-mediated enhanced infectivity of EBOV at equal levels (Fig. 3B), indicating that 5 aa, which was lost in TIM-1-359aa variant, was dispensable for interaction between TIM-1 and Ebola virus. We next determined whether TIM-1 ECD protein could prevent infection of EBOV and EBOV mutants bearing variant GPs. The inhibitory activity of TIM-1 ECD protein to all EBOV species tested was observed (Fig. 3C). Considering increased susceptibility of mutated Ebola virus species, recombinant TIM-1 protein has the potential to be a therapeutic avenue for Ebola virus infection.

Next, to determine whether the IgV domain mediates the function of TIM-1 protein, we prepared recombinant TIM-1 IgV (S21 to S121) and TIM-1 ΔIgV (L122 to T293, which excludes the IgV domain). The results showed that the blocking activity of TIM-1 IgV was comparable to that of TIM-1 ECD protein, while TIM-1 ΔIgV lost the activity to impede EBOV entry into TIM-1-expressing cell lines (Fig. 3D). In accord with this, TIM-1 ECD and TIM-1 IgV protein, rather than TIM-1 ΔIgV, could bind to PS (Fig. 3E), indicating that the IgV domain is critical for the inhibitory activity of TIM-1 protein.

**FIG 4:**
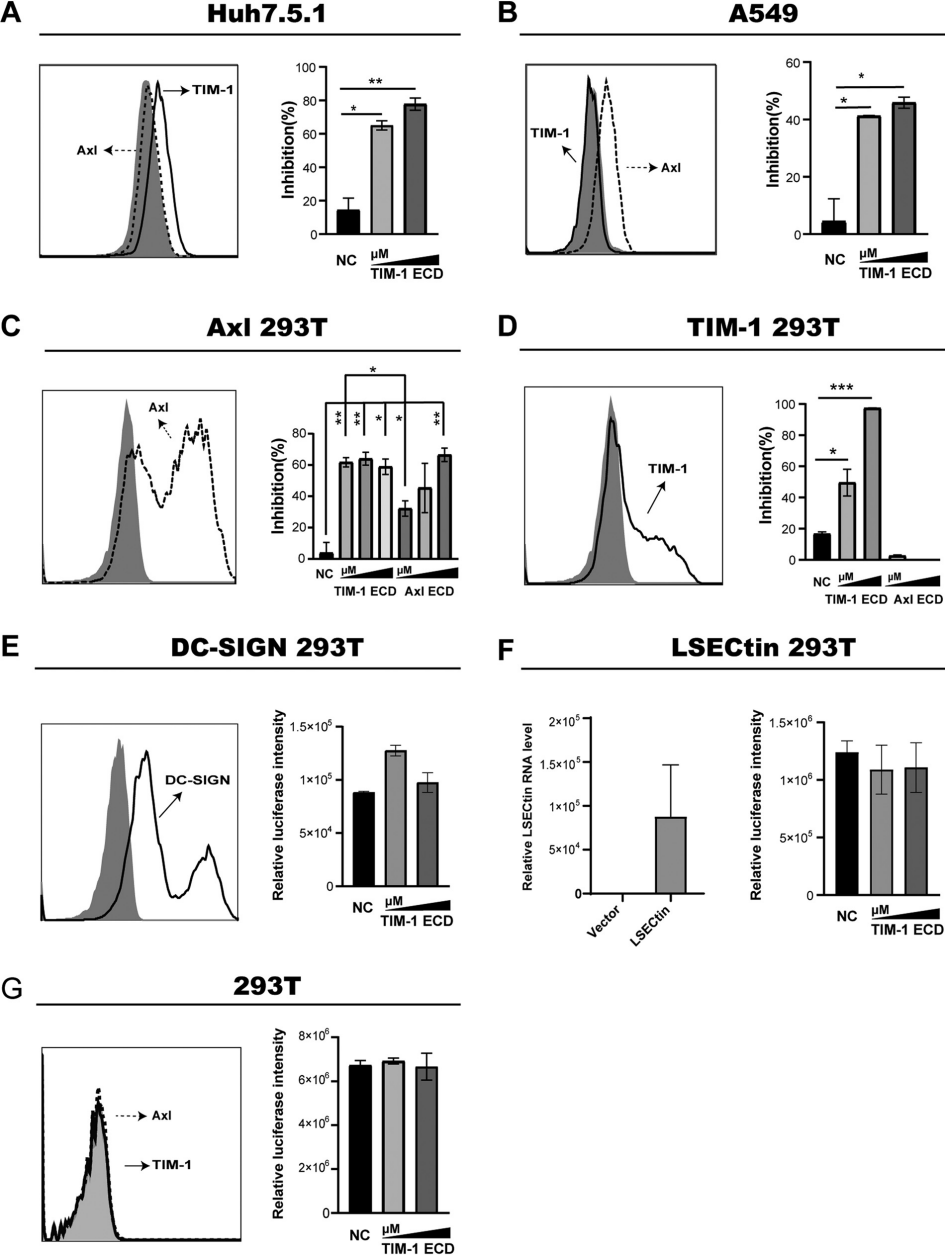
Inhibition of EBOV infection by TIM-1 ECD in different cell lines. (Left panel) Flow cytometric detection of TIM-1, Axl, and DC-SIGN expression on the surface of Huh7.5.1 cells (A), A549 cells (B), Axl-expressing 293T cells (C), TIM-1-expressing 293T cells (D), DC-SIGN-expressing cells (E), and 293T cells (G) using PE-conjugated anti-TIM, PE-conjugated anti-Axl, and APC-conjugated anti-DC-SIGN antibodies. The expression level of LSECtin in LSECtin-expressing 293T cells (F) was detected by RT-qPCR. (Right panel) TIM-1 ECD protein impeded EBOV entry into Huh7.5.1 cells (A), A549 cells (B), Axl-expressing 293T cells (C), TIM-1-expressing 293T cells (D), DC-SIGN-expressing cells (E), LSECtin-expressing 293T cells (F), and 293T cells (G). *, *P* < 0.05; **, *P* < 0.01; ***, *P* < 0.001.

We next investigated if TIM-1 competes for entry in a cell type (Huh7.5.1) that endogenously expressed TIM-1, but not Axl. TIM-1 ECD protein could impede the cellular entry of Ebola virus in this cell line (Fig. 4A). Intriguingly, TIM-1 protein also exhibited the inhibitory function on another cell line, which constitutively expressed Axl but not TIM-1 (Fig. 4B). To further address this, TIM-1 and Axl plasmids were introduced in the 293T cell line, respectively, and then the ability of TIM-1 ECD and Axl ECD proteins to compete for EBOV cell entry was tested. As expected, both TIM-1 ECD and Axl ECD protein displayed the inhibitory function in Axl-expressing cells (Fig. 4C). In sharp contrast, Axl ECD protein had no effects on competing for entry in TIM-1-expressing cells (Fig. 4D). Furthermore, although ectopic expression of DC-SIGN and LSECtin enhanced EBOV infection of 293T cells, TIM-1 protein did not inhibit LSECtin/DC-SIGN-mediated EBOV infection (Fig. 4E and F). Also, TIM-1 protein did not compete for viral entry into naive 293T cells, which was null for TIM-1 and Axl (Fig. 4G). These results indicated that TIM-1 protein potently inhibits the infection by Ebola virus of host cells that express PS receptor.

### TIM-1 protein synergizes with ADI-15946 to impede cell entry of Ebola virus.

**FIG 5:**
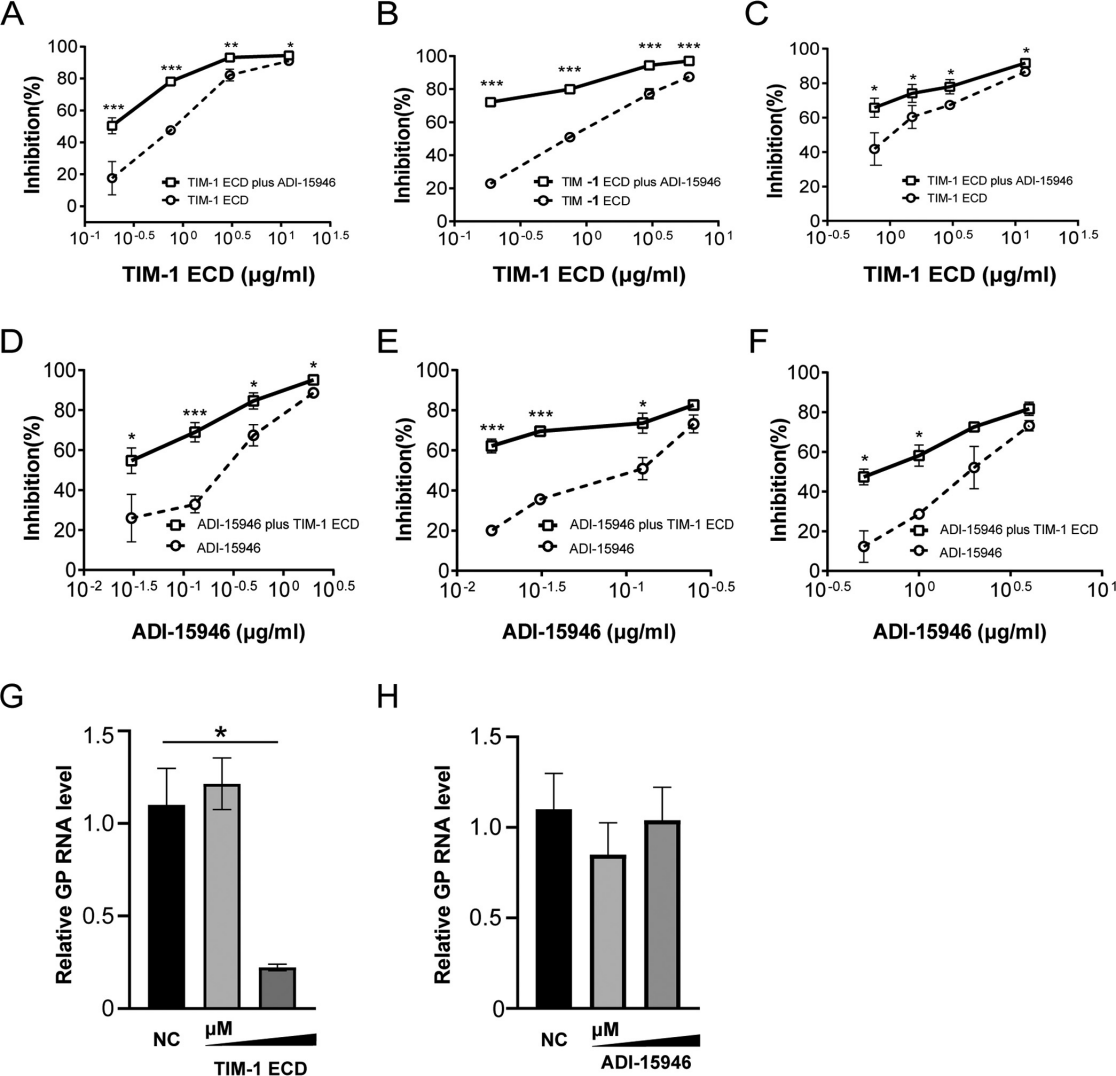
Analysis of synergistic effects of TIM-1 protein and ADI-15946 on impeding cell entry of virus. The inhibition was evaluated by varying one substrate at a fixed concentration of another. (A, B, C) Cooperative function of TIM-1 protein and ADI-15946 on impeding cell entry of EBOV (A), BDBV (B), SUDV (C) when the doses of TIM-1 ECD were titrated. (D, E, and F) Cooperative function when the doses of ADI-15946 were titrated. (G) TIM-1 ECD inhibits EBOV attachment, but ADI-15946 does not. EBOV pseudovirus was incubated with TIM-1 ECD (left panel) or ADI-15946 (right panel) at 37°C for 1 h before being added to TIM-1-expressing cells. Then, the cell-virus blend was transferred to 4°C for 30 min to allow virus attachment, and cells were washed with PBS three times. Total RNA was extracted and used for quantification of EBOV GP by RT-qPCR. *, *P < *0.05; **, *P* < 0.01; ***, *P* < 0.001.

Monoclonal antibodies targeting Ebola virus GPs have displayed superior efficacy in virus-infected animals and humans. ADI-15946 is a monoclonal antibody with broad activity for neutralizing three Ebola virus species (EBOV, BDBV, and SUDV). We first examined its binding capacity to Ebola virus GPs, which were prepared using a eukaryotic expression system. The high binding affinity of ADI-15946 to EBOV/BDBV GP was seen, while its engagement with SUDV GP was much worse (see Fig. S1A and B in the supplemental material). Accordingly, the inhibitory function of ADI-15946 on cell entry of EBOV and BDBV was much stronger than that on SUDV (Fig. S1C). Therefore, we wondered whether coadministration of TIM-1 protein and ADI-15946 is more efficacious than administration of either single agent alone. The results showed synergistic effects of TIM-1 protein and ADI-15946 on impeding cell entry of all virus species tested. Specifically, compared with TIM-1 protein treatment alone, combination with ADI-15946 dramatically enhanced inhibitory effects on the infectivity of all three virus species to cells (Fig. 5A to C). This effect was more significant in EBOV and BDBV. We also assessed the cooperative function of these two agents when the doses of ADI-15946 were titrated. Similar results were found (Fig. 5D to F). Interestingly, we found that TIM-1 ECD inhibited virus binding to TIM-1-expressing 293T cells, while ADI-15946 had no inhibition of TIM-1-mediated viral attachment (Fig. 5G). Taken together, these data suggest that coadministration of antibody and TIM-1 protein may represent a potential therapeutic regimen for Ebola virus treatment.

### TIM-1 protein potently blocks cell entry of antibody-evading Ebola virus species.

**FIG 6:**
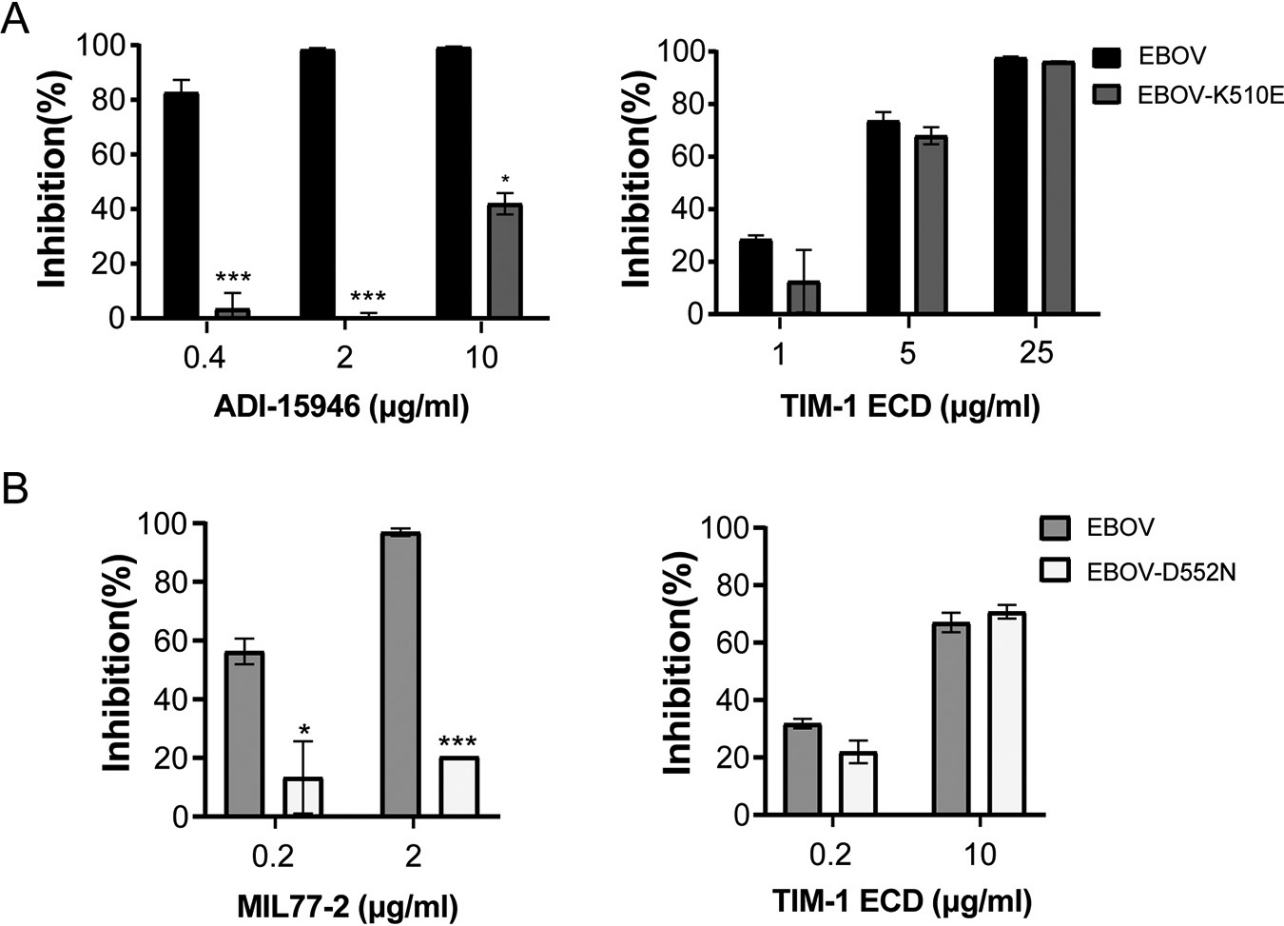
TIM-1 protein potently blocks cell entry of antibody-evading Ebola virus bearing GP mutations. (A) Inhibition of ADI-15946 (left panel) and TIM-1 ECD (right panel) to EBOV and EBOV bearing escape variant GP-K510E of ADI-15946. (B) Inhibition of MIL77-2 (left panel) and TIM-1 ECD (right panel) to EBOV and EBOV bearing escape variant GP-D552N of MIL77-2. *, *P* < 0.05; ***, *P* < 0.001.

Recently, several mutated Ebola virus species have been found to evade antibody attack (5–11). EBOV harboring GP-K510E and GP-D552N was refractory to ADI-15946 and MIL77-2 treatment, respectively (30, 36). We asked if these mutated species were sensitive to TIM-1 protein treatment. Although the neutralizing activity of ADI-15946 and MIL77-2 to mutated species was impaired pronouncedly, TIM-1 protein still exhibited excellent potency to impede the cell entry of these mutated species, which was equal to that of parental EBOV (Fig. 6A and B). This indicates strongly that TIM-1 protein may be an alternative therapeutic strategy to Ebola virus infection when antibody treatment is invalid.

## DISCUSSION

TIM-1 has been recently identified as one of the major attachment factors to facilitate entry of enveloped virus into host cells, including filovirus (15–20), flavivirus (24, 49–51), alphavirus (19, 51, 52), baculovirus (19), rhabdovirus (52), and New World arenavirus (51). Ectopic TIM-1 expression in poorly permissive cells dramatically enhanced EBOV infection, and silencing TIM-1 by RNA interference (RNAi) decreased infection of highly permissive cells (17). In EBOV-infected mice, loss of TIM-1 expression resulted in decreased virus load late during infection and significantly reduced virus-elicited mortality (16, 18).

In this study, we found that human TIM-1 plays a critical role in Ebola virus infection. Of the two major TIM-1 variants (TIM-1-359aa and TIM-1-364aa), TIM-1-359aa was a natural variation of TIM-1-364aa with amino acid substitutions (L to P) and deletion (157del MTTVP) within the MLD site (20). It is worth noting that these two TIM-1 variants have been demonstrated to play different roles in enhancing infectivity (25–29). TIM-1-364aa overexpression significantly promoted the entry and infection of Japanese encephalitis virus compared with the short form of TIM-1 (25). In human immunodeficiency virus, homozygosity for the short allele of TIM-1 was associated with natural protection from infection and a lower rate of virus replication in CD4^+^ T lymphocytes (28). Additionally, TIM-1-364aa was associated with more severe outcome of infection with hepatitis A virus (26, 27). In this study, we demonstrated that these two variants promoted Ebola virus attachment, internalization, and infection, with no significant difference in magnitude. The relationship between TIM-1 polymorphism and virus infection needs further investigation.

EBOV strains harboring GP mutations (A82V and T544I) have been shown to reduce the stability of the prefusion conformation of GP compared to their parental strain and thus enhance infection by decreasing the threshold for activation of membrane fusion activity triggered by host factors cathepsin B and Niemann-Pick C1 (6, 7, 53). Also, some researchers attributed the enhanced infection to I544 stabilizing the primed GP structure by increasing the hydrophobicity around residue 544, which is critical for virus-host membrane fusion (9, 54, 55). We showed that EBOV harboring point mutations A82V and A82V T544I of GP had stronger sensitivity to TIM-1/Axl- but not DC-SIGN/LSECtin-mediated entry than wild-type virus. This can be explained by the difference in the magnitude of PS anchored on the envelope of virus species studied here. Nanbo et al. demonstrated that GP plays a role in affecting the topology of PS in Ebola virus virions, which is mediated by recruiting a scramblase to the viral particles (56). Therefore, it appears conceivable that the point mutation A82V on GP may lead to the alteration of scramblase trafficking and an increase in PS levels and ultimately augment TIM-1/Axl-mediated Ebola virus entry. The details need to be elucidated in the future.

Given the definitely infection-promoting role of TIM-1, its ectodomain has been considered an antagonist to reduce virus infectivity (17, 24). Transfection of TIM-1-Fc-expressing plasmid into the TIM-1-positive H3 cell line led to a significant reduction of EBOV GP-dependent infection (17). We developed TIM-1 ECD protein that inhibits PS-dependent infection and showed that TIM-1 ECD can block infection of wild-type Ebola virus, as well as those antibody-refractory species.

Furthermore, TIM-1 protein also impeded the entry of virus into TIM-1-null A549 host cells, which expressed Axl, indicating that TIM-1 protein has potentials to disrupt interaction between Ebola virus and other PS receptors—for example, TAM receptors (40–44). Conversely, Axl ECD protein treatment was not effective in inhibiting TIM-1-mediated viral entry, most likely because of the higher affinity of TIM-1 for PS binding compared to Axl (48). These results suggest that TIM-1 may represent a therapeutic avenue for Ebola virus infection, especially for PS-dependent Ebola virus entry. Our study here further extends these findings and provides evidence that TIM-1 is also utilized by EBOV with various residue changes of GP to enhance infectivity. We show that in spite of resistance to antibody treatment, EBOV strains harboring GP mutations (K501E and D522N) are still sensitive to TIM-1 protein-mediated inhibition of infection. This may be particularly important for urgent administration of TIM-1 protein in case of an outbreak of these antibody-resistant species. Further studies are warranted to determine the inhibitory activity of TIM-1 protein on these antibody-refractory Ebola virus species *in vivo*.

Overall, this study uncovered an important role of TIM-1 in promoting Ebola virus infection and demonstrated that TIM-1 protein may be a potential antagonist to reduce Ebola virus-elicited tissue damage. The investigations on mechanistic details in TIM-1-mediated enhancement of infectivity of Ebola virus are ongoing. We will utilize EBOV-infected animals to evaluate the function of TIM-1 protein in the future to determine whether the effects of a pharmacological approach to block TIM-1 are comparable to those of the phenotype of TIM-1 knockout mice subjected to EBOV infection.

## MATERIALS AND METHODS

### Cells and plasmids.

Human embryonic kidney cells of the 293T line were purchased from the Chinese Academy of Sciences. Human lung adenocarcinoma cells of line A549 and human hepatoma cells of line Huh7.5.1 were kept by our laboratory. All cells were all grown in Dulbecco’s modified Eagle’s medium (DMEM) (Gibco) supplemented with 10% fetal bovine serum (Gibco), penicillin, and streptomycin (Gibco) in a humidified atmosphere (5% CO_2_, 95% air) at 37°C in an incubator. The VSV-ΔG* vesicular stomatitis virus stock, EBOV (AMT75575) or (A0A068J419) and mutated EBOV, BDBV (AYI50382) or (B8XCN0), and SUDV (Q7T9D9) GPs and HIV-based vector pSG3.Δenv.cmvFluc plasmids were kind gifts from the China Institute for Food and Drug Control. The VSV-G plasmid was purchased from YouBia Biology (VT1470). The Axl (P30530) and TIM-1-359aa plasmids were purchased from Sino Biological. TIM-1-364aa (Q96D42), DC-SIGN (Q9NNX6), and LSECtin (Q6UXB4) plasmids were synthesized by Genewiz. TIM-1-N114A D115A plasmid was obtained by overlap PCR.

### Pseudovirus preparation and titer.

HIV bearing Ebola virus and VSV GPs were generated by liposome-mediated transfection system (Polyplus; 25Y1801N5) as follows. 293T cells were seeded into 6-well plates at 7 × 10^5^ cells per well and transfected with a total of 2 μg plasmid DNA when cells yielded a density of 60 to 80% confluence. Amounts of 0.4 μg GP or VSV-G and 1.6 μg pSG3.Δenv.cmvFluc were utilized according to the optimized results. The cell supernatants were harvested 48 h after transfection, centrifuged at 3,000 rpm for 10 min, filtered through a 0.45-μm-pore filter, and frozen in aliquots at −80°C. Titers of the pseudovirus were determined by an HIV p24 ELISA kit (Key-Bio, K12P2401).

### Pseudovirus entry and neutralization assay.

TIM-1-, Axl-, DC-SIGN-, and LSECtin-expressing 293T cells were obtained by transfection of 293T cells with corresponding plasmids by using a liposome-mediated transfection system. For infections, 200 μL of dilutions of pseudovirus was added to 96-well plates at 3 × 10^4^ cells/well. Thirty-six to 48 h postinfection, expression levels of firefly luciferase were detected by a fluorescence microplate reader (Promega). Briefly, 100 μL liquid from each well was aspirated and discarded, followed by the addition of 100 μL Bright-Glo (Promega, E6120) luciferase substrate. Two minutes after the reaction mixture was shielded from light, 150-μL mixtures were transferred to the 96-well whiteboards and detected. For the neutralization assay, pseudoviruses were incubated with dilutions of ADI-15946 or TIM-1 protein in duplicate at 37°C for 1 h before being added to TIM-1-expressing 293T cells. To characterize cooperative neutralization of the ADI-15946 and TIM-1 simultaneously, we assessed the inhibition of TIM-1 protein or ADI-15946 in the presence or absence of the other. For the annexin V-associated inhibition assay, EBOV was incubated with annexin V (Acro; AN5-H5118) in 1× binding buffer at 37°C for 1 h before being added to TIM-1-expressing 293T cells.

### Viral attachment and internalization assay.

293T and TIM-1-expressing 293T cells were seeded into 12-well plates (4 × 10^5^ cells/well) overnight. Then, the culture medium was discarded, the EBOV pseudovirus was added, and the cells were incubated with EBOV at 4°C for 30 min (attachment assay). After being washed with phosphate-buffered saline (PBS) three times, the unbound EBOV was removed, and total RNA was extracted and used for quantification of the EBOV level by reverse transcription quantitative PCR (RT-qPCR). For the internalization assay, cells were incubated with EBOV at 4°C for 1 h, washed with PBS, and then shifted to 37°C for 2 h. Uninternalized virions were removed by 1 mg/mL proteinase K (CWbio; CW2584), and total RNA was extracted (25, 50). For the attachment inhibition assay, the TIM-1 ECD protein and ADI-15946 were first incubated with EBOV at 37°C for 1 h, and then the blend was added to TIM-1-expressing 293T cells and shifted to 4°C for 30 min. Washing and extraction steps were performed as described above.

### Real-time PCR.

The total RNA in EBOV-infected, LSECtin-expressing 293T cells was extracted by the Animal Total RNA isolation kit (Foregene; RE-03014), and cDNA was obtained from RNA using the EasyQuick RT MaterMix (Cwbio; CW2019). The level of mRNA was determined by RT-qPCR, using MagicSYBR mixture (Cwbio; CW3008) and specific primers in the real-time PCR system (Applied Biosystems QuantStudio 3&5; Invitrogen). The primers for EBOV GP, TIM-1, GAPDH (glyceraldehyde-3-phosphate dehydrogenase), and LSECtin mRNA quantification were as follows: E-Forward (ACTCATCACCAAGATACCGGA) and E-Reverse (GTTATGTTCTTGGTCCAATCA), which are complementary to EBOV sequence; primers TIM-1 T-Forward (ATGCATCCTCAAGTGGTCATC) and T-Reverse (TTAGTCCGTGGCATAAAGACTAT); primers GADPH G-Forward (AGGTCGGTGTGAACGGATTTG) and G-Reverse (TGTAGACCATGTAGTTGAGGTCA); and primers LSECtin L-Forward (CAATGCCTCCAAACAGACCG) and L-Reverse (TGAACAGCTCGGTTCTCACG). Relative gene expression was calculated based on the comparative threshold cycle (*C_T_*) method, using GAPDH as an internal reference.

### Western blots.

293T and TIM-1-expressing 293T cells were seeded into 6-well plates (7 × 10^5^cells/well) overnight. Then, the same amount of pseudovirus was added to these cells. After 48 h, cells were washed three times with PBS and then lysed in radioimmunoprecipitation assay (RIPA) buffer (Cwbio; CW2333) supplemented with protease inhibitor cocktail (Cwbio; CW2200) for 30 min on ice. Lysis products were then subjected to centrifugation. After centrifugation, 10 μL supernatants separated from each sample was loaded per lane using 10% SDS-PAGE and transferred to a polyvinylidene difluoride (PVDF) membrane (Merck; IPVH00010). After blocking with 5% nonfat milk, the membrane was incubated with the primary antibodies anti-EBOV mab114, anti-TIM-1, and anti-GAPDH (CST; 88884S) overnight at 4°C. Subsequently, the membrane was incubated with horseradish peroxidase (HRP)-conjugated anti-human (Invitrogen; A18817) and anti-rabbit (Zsgb-Bio; ZB2301) secondary antibodies for 45 min at room temperature. Immunoreactivity was detected using an enhanced chemiluminescence detection system (Chemiscope 6000; CLiNX). For p24 and GP assembly detection, pseudovirus supernatants were loaded directly using 10% SDS-PAGE; p24 primary antibody was purchased from (R&D; MAB73601-100).

### Flow cytometry.

293T cells were transfected with 0.5 μg, 1.0 μg, and 2 μg of TIM-1-364aa and 2 μg of TIM-1-359aa and empty vector pcDNA3.1(+) expression plasmids. Thirty hours later, cells were harvested and incubated with phycoerythrin (PE)-conjugated anti-TIM-1 (R&D; FAB1750P) for 30 min at 4°C. Cells were washed extensively, and TIM-1 expression was detected on a FACSAria II flow cytometer (BD Biosciences). Data analysis was performed using the FlowJo software. The expression of Axl and DC-SIGN was detected by PE-conjugated anti-Axl (R&D; FAB154P) and allophycocyanin (APC)-conjugated anti-DC-SIGN (Biolegend; 330107) as described above.

### Enzyme-linked immunosorbent assay.

For detection of direct interactions between Ebola virus GPs and ADI-15946, His-tagged GPs 2 μg/ml were first coated with coating buffer (0.1 M Na_2_CO_3_ and NaHCO_3_ [pH 9.6]) on 96-well plates overnight at 4°C. Wells were washed with PBST (PBS plus 0.2% Tween 20) and saturated for 1 h at 37°C with PBS–4% nonfat milk. Three-fold dilutions of ADI-15946 were added, and the mixture was incubated for 1 h at 37°C. Bound particles were detected with HRP-conjugated goat anti-human IgG antibody (Invitrogen; A18817). PS-associated ELISAs were carried out as previously described (57). PS (Sigma; P7769) and phosphatidylcholine (PC) (Sigma; P3556) were first dissolved in chloroform as a stock solution. Then, the solution was diluted to 5 μg/mL with methanol and added to the ELISA plate. Upon being dried, the plates were blocked with Tris-buffered saline (TBS)–4% bovine serum albumin (BSA) fraction V. TIM-1 ECD, TIM-1 IgV, TIM-1 ΔIgV, and control protein were diluted in TBS–10 mM CaCl_2_, and the mixture was added to the wells and incubated at 37°C for 1 h. The plates were washed with TBST (TBS plus 0.05% Tween 20) before incubation with HRP-conjugated mouse anti-human His antibody (Biolegend; 652503). The absorbance at 450 nm was measured by microplate reader (Thermo Fisher Scientific).

### Binding kinetics assay.

The binding kinetics and affinity of Ebola virus GPs to antibody ADI-15946 were measured using the ForteBio biofilm interferometry technique. Briefly, the AHC biosensors targeting IgG Fc tips were prewet with PBST (PBS plus 0.02% Tween 20). The following approach was used, summed up in 6 steps: baseline, loading, baseline, association, dissociation, and regeneration. Biosensors were dipped in PBST first to establish a test baseline, and then antibody ADI-15946 (8 μg/mL) was loaded. The biosensors loaded with antibody were immersed with different concentrations of GPs to determine the association constant and then transferred into PBST to determine the dissociation constant. In the end, the biosensors were eluted with 10 mM glycine HCl (pH 1.7) to regenerate. The results were analyzed by ForteBio version 9.0.0.4 data analysis software to determine the equilibrium dissociation constant, KD.

### Antibodies and protein preparation and purification.

Ebola virus GPs lacking the mucin domain and transmembrane domain (EBOV, aa 33 to 632, Δ312-464; BDBV, aa 33 to 640, Δ313-470; SUDV, aa 34 to 650, Δ320-476), the TIM-1 ectodomain (S21 to T293), the IgV domain of TIM-1 (S21 to S121), and TIM-1 ΔIgV (L122 to T293) with a histidine tag at the C terminus were synthesized and subcloned into an optimized mammalian expression vector. The VH and VL sequence of antibodies were synthesized and cloned to a mammalian full-length immunoglobulin expression vector. Purification was performed using the ÄKTAprime Plus system (GE Healthcare). Concentrations were quantified using the bicinchoninic acid (BCA) method.

### Statistical analyses.

Graphical and statistical analyses were performed using Prism software (GraphPad). Data are presented as mean ± standard error (SE) values according to multiple independent experiments. Unpaired two-tailed *t* tests were used to compare experiment and control groups. *P* values of <0.05 were considered statistically significant.
