# Supplementary material for: TIM-1 Augments Cellular Entry of Ebola Virus Species and Mutants, Which Is Blocked by Recombinant TIM-1 Protein
Source: Microbiol Spectr. 2022 Apr 6;10(3):e02212-21. doi: 10.1128/spectrum.02212-21 (PMC9241846; doi:10.1128/spectrum.02212-21)
Supplement: SUPPLEMENTAL FILE 1 — Supplemental material. Download spectrum.02212-21-s001.pdf, PDF file, 0.3 MB [file spectrum.02212-21-s001.pdf]

FIG S1 Antibody ADI-15946 related binding experiments. Analysis of the binding affinity of ADI-15946 with ebola virus GPs by (A) Fortebio and (B) *ELISA* methods. (C). Inhibiton ebola virus cell entry by ADI-15946.

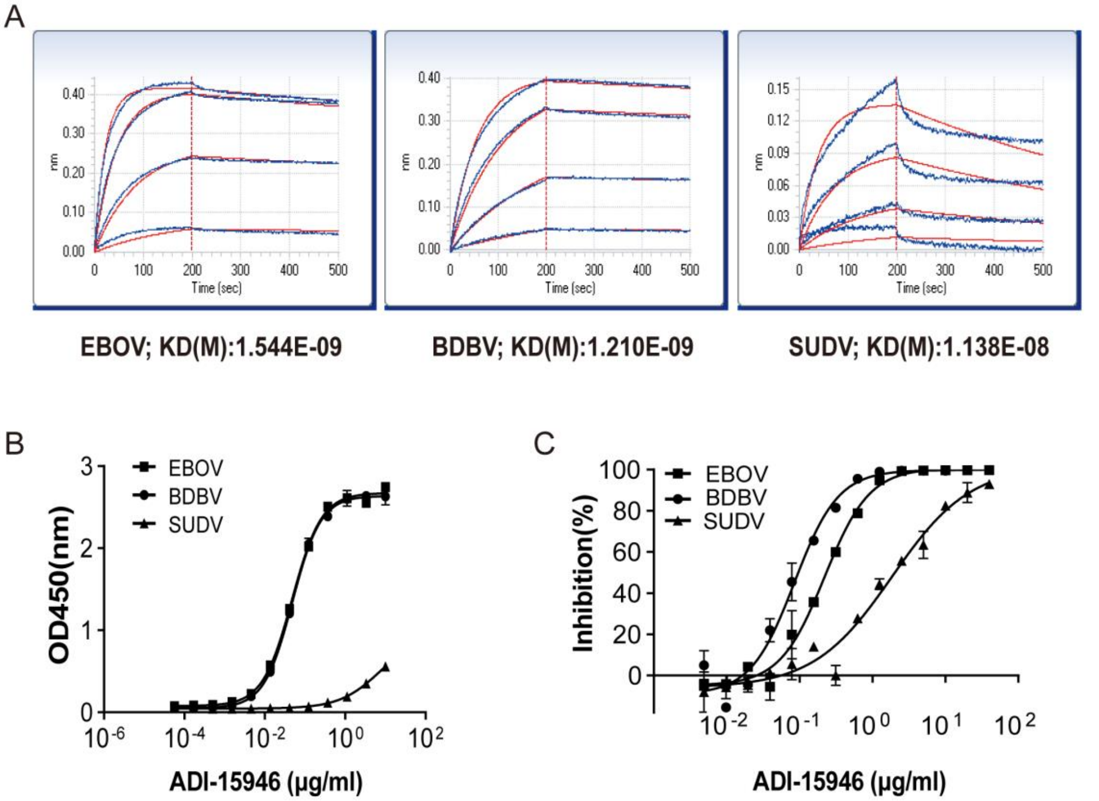

FIG S2 (A) Luciferase expression levels of pSG3.Δenv.cmvFluc-infected TIM-364aa-expressing 293T cells versus vector-expressing 293T cells, data are relative luciferase intensity.

A

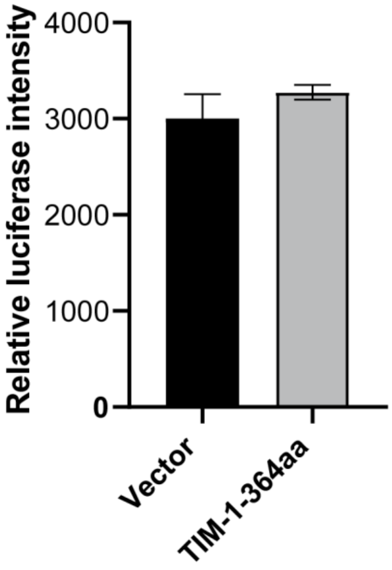
Relative luciferase intensity
4000
3000
2000
1000
0
Vector
TIM-1-364aa
